# Domestic violence in indian women: status of husbands’ alcohol consumption as a determinant factor

**DOI:** 10.1093/eurpub/ckac130.138

**Published:** 2022-10-25

**Authors:** J da Silva Maia, J Félix China, JP Teixeira

**Affiliations:** 1 Public Health Unit, Primary Healthcare Cluster Estuário do Tejo, Lisbon, Portugal; 2 Public Health Unit, Primary Healthcare Cluster Arrábida, Setúbal, Portugal; 3 Public Health Unit, Primary Healthcare Cluster Sintra, Lisbon, Portugal

## Abstract

**Background:**

Domestic violence continues to be a major public health issue that affects millions of individuals worldwide. According to the 2015-2016 India National Family Health Survey, 33% of married women had been victims of spousal physical, sexual, or emotional violence. It was also revealed that spousal abuse differs depending on the level of the husband's alcohol consumption. In this case, determining the link between this consumption and domestic violence is critical.

**Methods:**

The effects of husbands’ alcohol use on the occurrence of domestic violence and associated risk factors were investigated using data from the 2015-2016 India Individual Record Database (DHS) A binomial logistic regression model was used for the analysis.

**Results:**

62554 married women aged 15 to 49 were chosen for this study, of which 31.2% have experienced some form of marital violence. Women with husbands who drink alcohol account for 31% of the sample, with 49.5% of those committing domestic violence against their wives. In a multivariate analysis, women whose spouses drink have a 3.11 times higher chance (p < 0.01; adjOR=3.11, 95%CI 3-3.23) of experiencing domestic violence than women whose husbands don't drink.

**Conclusions:**

The effect of the husband's alcohol use on the occurrence of domestic violence can be used to guide evidence-based targeted intervention.

**Key messages:**

- Domestic violence is a public health issue with potential for targeted intervention.
- Targeted interventions in husbands’ alcohol consumption of women who suffer domestic violence might guide useful interventions to address this problem.

